# Convalescent *Plasmodium falciparum*-specific seroreactivity does not correlate with paediatric malaria severity or Plasmodium antigen exposure

**DOI:** 10.1186/s12936-018-2323-4

**Published:** 2018-04-25

**Authors:** Anne Kessler, Joseph J. Campo, Visopo Harawa, Wilson L. Mandala, Stephen J. Rogerson, Wenzhu B. Mowrey, Karl B. Seydel, Kami Kim

**Affiliations:** 1 Albert Einstein College of Medicine, Bronx, NY USA; 2 Antigen Discovery Inc, Irvine, CA USA; 3 Malawi-Liverpool Wellcome Trust Clinical Research Programme, Blantyre, Malawi; 4 College of Medicine, Biomedical Department, University of Malawi, Blantyre, Malawi; 5 Academy of Medical Sciences, Malawi University of Science and Technology, Thyolo, Malawi; 6 The University of Melbourne, Melbourne, Australia; 7 College of Osteopathic Medicine, Michigan State University, East Lansing, MI USA; 8 Blantyre Malaria Project, University of Malawi College of Medicine, Blantyre, Malawi; 9 Morsani College of Medicine, University of South Florida, Tampa, FL USA

**Keywords:** *Plasmodium falciparum*, Antibody, Protein microarray, Cerebral malaria, Uncomplicated malaria, PfEMP1

## Abstract

**Background:**

Antibody immunity is thought to be essential to prevent severe *Plasmodium falciparum* infection, but the exact correlates of protection are unknown. Over time, children in endemic areas acquire non-sterile immunity to malaria that correlates with development of antibodies to merozoite invasion proteins and parasite proteins expressed on the surface of infected erythrocytes.

**Results:**

A 1000 feature *P. falciparum* 3D7 protein microarray was used to compare *P. falciparum*-specific seroreactivity during acute infection and 30 days after infection in 23 children with uncomplicated malaria (UM) and 25 children with retinopathy-positive cerebral malaria (CM). All children had broad *P. falciparum* antibody reactivity during acute disease. IgM reactivity decreased and IgG reactivity increased in convalescence. Antibody reactivity to CIDR domains of “virulent” PfEMP1 proteins was low with robust reactivity to the highly conserved, intracellular ATS domain of PfEMP1 in both groups. Although children with UM and CM differed markedly in parasite burden and PfEMP1 exposure during acute disease, neither acute nor convalescent PfEMP1 seroreactivity differed between groups. Greater seroprevalence to a conserved Group A-associated ICAM binding extracellular domain was observed relative to linked extracellular CIDRα1 domains in both case groups. Pooled immune IgG from Malawian adults revealed greater reactivity to PfEMP1 than observed in children.

**Conclusions:**

Children with uncomplicated and cerebral malaria have similar breadth and magnitude of *P. falciparum* antibody reactivity. The utility of protein microarrays to measure serological recognition of polymorphic PfEMP1 antigens needs to be studied further, but the study findings support the hypothesis that conserved domains of PfEMP1 are more prominent targets of cross reactive antibodies than variable domains in children with symptomatic malaria. Protein microarrays represent an additional tool to identify cross-reactive *Plasmodium* antigens including PfEMP1 domains that can be investigated as strain-transcendent vaccine candidates.

**Electronic supplementary material:**

The online version of this article (10.1186/s12936-018-2323-4) contains supplementary material, which is available to authorized users.

## Background

Antibody immunity to *Plasmodium falciparum* malaria is central to the prevention and clearance of symptomatic disease [1], but is acquired only after years of exposure [2]. Immunity to malaria is non-sterile, and older children and adults in malaria-endemic areas are frequently parasitaemic, but asymptomatic. Children in sub-Saharan Africa carry the burden of morbidity and mortality associated with *P. falciparum* infection [3]. It is still unclear why some parasitaemic children develop severe malarial syndromes, including cerebral malaria (CM).

Although not all studies agree [4], prior studies have reported ‘protective’ antibody responses that differentiate asymptomatic cases from symptomatic malaria [5–7]. Most studies have found few differences in antibody profiles along the symptomatic disease spectrum [8–10], but tested a limited number of antigens. Newer technologies, including protein microarrays, provide an alternative platform for large-scale evaluation of seroreactivity to thousands of antigens [11]. An initial large *P. falciparum* proteome-wide array encompassed approximately a quarter of the inferred proteome (2320 proteins encoded by 1204 genes) and identified 491 immunoreactive proteins recognized by sera from a cohort in Mali [12]. A subsequent 824 feature array representing 699 Pf genes was down-selected on the basis of initial array data [5].

In CM, *P. falciparum* erythrocyte membrane protein 1 (PfEMP1), a parasite variant surface antigen (VSA) encoded by ~ 60 *var* genes, is critical for cerebral sequestration [13, 14]. Since antibodies to VSA correlate to protection from symptomatic malaria [10], children who progress to CM may lack antibodies targeting antigens essential for microvasculature sequestration. Each PfEMP1 protein is comprised of multiple extracellular Duffy-binding like (DBL) and cysteine-rich interdomain region (CIDR) domains that are classified into sequence types (e.g. α, β, γ) and subtypes (e.g. CIDRα1) [15, 16]. The N-terminal head structure (DBL-CIDR) of each PfEMP1 protein is a determinant of PfEMP1-host binding specificity. CIDRα2–6 domains encode CD36 binding properties, and CIDRα1 domains encode endothelial protein C receptor (EPCR) binding properties. CIDRβ/γ/δ domains have unknown binding properties but have been associated with rosetting, which occurs when parasitized red blood cells (pRBCs) bind and aggregate uninfected RBCs [17]. Studies of paediatric malaria implicate EPCR-binding parasites in severe disease [18–25]. Breadth and magnitude of PfEMP1 seroreactivity correlates with age and exposure [26, 27], and antibodies to EPCR-binding CIDRα domains are more abundant than antibodies to other CIDR domains and are likely acquired early in life [28].

Here, partial proteome microarrays were used to identify and characterize differences in global antibody level, breadth and magnitude of *P. falciparum* antibody responses, and magnitude of PfEMP1 specific antibody responses between children with uncomplicated malaria (UM) and children with stringently defined CM (Ret + CM; WHO definition + malarial retinopathy) in acute infection and at 30 days of convalescence. A new ~ 1000 feature partial proteome array based upon the 3D7 *P. falciparum* reference genome that prioritized antigens with relevance to diagnostics and human anti-malarial immunity was used. The antigens represented on this array include vaccine candidates as well as proteins that have been associated with either exposure to *P. falciparum* or protection from clinical disease. The study results indicate that children with UM and CM have broad seroreactivity to the panel of ~ 1000 *P. falciparum* antigens and have lowest seroreactivity during acute disease to EPCR-binding “virulent” PfEMP1 antigens represented on the array [17, 25]. Despite exposure to “virulent” PfEMP1 during acute disease, antibody responses to the corresponding antigen in convalescence were not detected using this platform that was generated based upon the reference 3D7 genome.

## Methods

### Patient recruitment and sample collection

During the 2015–2016 malaria seasons, children with malaria at Queen Elizabeth Central Hospital (QECH) in Blantyre, Malawi were recruited to the study (see Additional file 1). UM cases were recruited from the Accident and Emergency Department and included children ages 1–12 years with fever, Blantyre coma score of 5, peripheral *P. falciparum* parasitaemia, and no overt signs of chronic disease, malnutrition, or progression to severe malaria; all were treated as outpatients. Children with CM were admitted to the Paediatric Research Ward at QECH with clinical, WHO-defined cerebral malaria (parasitaemia, Blantyre coma score ≤ 2, no other identifiable causes of coma) and were 6 months–12 years in age. Only children with CM with malarial retinal abnormalities (Ret + CM) were included. HIV + children were excluded. Cases reported in this study are a subset of those previously reported [25]. Of 38 UM and 57 ret + CM cases, cases were selected for inclusion if they met all of the following criteria: (1) attended 30 day follow-up appointment, (2) HIV non-reactive, (3) successful *var* qRT-PCR typing from acute infection, and (4) sufficient acute and convalescent plasma for the study. Comparable numbers of cases from each group were compared. Age and sex distribution were comparable to the larger group [25] and were not statistically different between the CM and UM group (Table 1).

**Table 1:** Clinical characteristics of Ret + CM and UM cases

Patient characteristic	Ret + CM (n = 25)	UM (n = 23)	P-value*
Age (yr), median [IQR]	3.5 [3, 4]	4 [2, 7]	0.29
Male, n (%)	15 (60.0)	17 (73.9)	0.31
Parasite smear score, median [IQR]	2 [2, 4]	4 [3, 4]	*0.01*
Pfhrp2 (ng/ml), median [IQR]	2580 [566, 10, 506]	216 [57, 484]	*<* *0.001*
Cell free Pf DNA (genomes per μl), median [IQR]	904 [414, 1703]	60 [5, 471]	*<* *0.001*
Hgb (g/dL), median [IQR]	7.4 [6.4, 9.1]	9.6 [7.7, 11.5]	*0.01*
Platelets (10^3^/μl), median [IQR]	66 [49, 88]	126 [64, 153]	*0.03*
Total WBC (10^3^/μl), median [IQR]	8.8 [6.5, 11.9]	7.8 [6.0, 9.4]	0.20
Var targets amplified^a^ (of n = 48), median [IQR]	21 [17, 24]	11 [7, 17]	*<* *0.001*
ICAM-1 motif amplified^b^, n (%)	10 (52.6%)	5 (21.7%)	*0.04*
Mortality, n (%)	0 (0)	0 (0)	1.00

IQR, interquartile range; * P-values correspond to Wilcoxon rank-sum or Chi squared test

^a^N = X targets total, amplified using primers from Lavstsen et al. [19], Mkumbaye et al. [44]

^b^Motif amplified using primers from Lennartz et al. [32]; Ret + CM n = 19

### Quantification of plasma PfHRP2 levels

PfHRP2 was quantified in patient plasma by ELISA [29]. Samples were diluted using phosphate-buffered saline and plated in duplicate onto plates coated with anti-HRP2 antibody (Cellabs, Brookvale, Australia). Recombinant PfHRP2 was used to generate a standard curve for quantification. Patient samples with PfHRP2 outside of the linear range of the standard curve were diluted further and reanalysed.

### Direct quantification of parasite DNA in plasma

Cell-free, parasite DNA was measured directly from acute patient plasma by qPCR using a primer–probe set targeting *Plasmodium* 18S rRNA [30]. Amplifications were performed in duplicate on an Applied Biosystems 7300 PCR system. Standard curves were generated from harvested parasite cultures of known parasitaemia and used to calculate parasite genomes in plasma samples.

### Determination of total Immunoglobulin plasma concentrations

Total plasma IgG and IgM were measured in acute and follow-up (FU) samples using human isotype-specific radial immunodiffusion kits (The Binding Site Ltd.). Plasma was heat-killed prior to use, and all samples were assayed in duplicate.

### Protein microarray development and chip design

A partial proteome microarray with 1000 *P. falciparum* protein features (Pf1000, 3rd generation) was developed at Antigen Discovery, Inc. (ADI, Irvine, CA, USA). Proteins were selected based on internal R&D using *P. falciparum* full proteome microarrays probed with samples collected from subjects during naturally and experimentally acquired immunity (unpublished). Briefly, proteins were selected based on a prioritization scheme as follows: (1) a curated list of *P. falciparum* proteins prominently featured in the scientific literature and established targets of *P. falciparum* candidate vaccines and diagnostics, plus the full repertoire of 3D7 PfEMP1, rifin and stevor proteins, (2) top hits from previous studies with protein arrays that identify correlates of protection or exposure, (3) a ranking of proteins based on ADI internal full proteome microarray data. The criteria for ranking proteins with empirical full proteome data employed a scoring system that prioritized: seroprevalence in either naturally immune populations (> 60%) or pre-erythrocytic experimental immunity (> 20%); predicted transmembrane domains; predicted signal peptides; gene ontology (GO) terms for Function, Process and Component that were broadly related to parasite and host cell surfaces, host-parasite interactions and host immune system. Proteins were selected according to the prioritization scheme until a total of 1000 targets were selected, accounting for fragmented proteins. The 1000 full-length or partial *P. falciparum* proteins represent 762 genes from *P. falciparum* reference strain 3D7, including 61 PfEMP1s, vaccine candidate proteins, and 176 conserved *Plasmodium* proteins of unknown function. Protein microarrays were fabricated as described in Additional file 2.

### Protein microarray sample probing

Antibody reactivity (log_2_ scale) was determined relative to signal of expression system controls (i.e. “Background”), with signal intensity greater than or equal to 2× the background signal considered seropositive [31] (i.e. R ≥ 1). Antibody breadth, represented as breadth scores, was calculated as the sum of seropositive responses per individual. Antibody magnitude (levels) was defined as the log_2_-transformed ratio of target signal to background signal, represented by the normalized signal intensity. Antibody “Deltas” were calculated by subtracting normalized antibody levels at 30-day convalescence from acute antibody levels. Samples were probed and analyzed as described in Additional file 2.

### PfEMP1 specific annotation and analysis

Pf1000 protein microarray PfEMP1 antigen spots were annotated using VarDom 1.0 Server [15] to predict domain and total protein architecture. Each PfEMP1 on the array was annotated for gene group, encoded domains and domain cassettes, the presence of the ATS (acidic terminal sequence), and binding phenotype (EPCR, CD36, ICAM-1, or rosetting) [17, 18, 32, 33]. For PfEMP1 proteins, the head structure, C terminal extracellular domains, and ATS were arrayed in separate spots for each PfEMP1, enabling independent assessment of antibody reactivity. PfEMP1 domain annotations for Pf1000 antigens (e.g. spots) on the array are outlined in Additional file 3. All annotations correspond to the DBLα-CIDRα head structure domains except for the ICAM-1 binding domains. *var* gene expression was determined by qRT-PCR from the peripheral blood using a panel of PfEMP1 specific primers [25] with an expression T_u_ value > 8 indicating positive expression [19].

### Control serum

Hyperimmune IgG and naïve sera were probed on the array as controls. Hyperimmune IgG was pooled from 834 HIV and HBsAg seronegative Malawian adults [34], and the naïve control was pooled from 30 malaria-naïve adults from the United States. Both controls were probed at 1:100 (20% *Escherichia coli* lysate; GenScript). Following dilution, the antibody concentration of the hyperimmune serum was 100 μg/ml.

### Statistical analysis

Statistical analysis was performed using Stata 12.1 or Prism 7.0 for Macintosh. Statistical differences were determined by Wilcoxon rank sum (continuous, unpaired variables), Wilcoxon signed rank sum (continuous, paired variables) or Chi squared test (categorical variables). Analysis of *P. falciparum* antibodies was performed in R version 3.1.1 [35] and Graphpad Prism (GraphPad Software, La Jolla California, USA), as detailed in Additional file 2. Statistical significance was assessed using an α level < 0.05. P values from multiple comparisons were corrected by the false discovery rate approach (FDR) [36].

## Results

### Children with CM have high parasite burden and clinical measures reflecting severe disease

**Fig. 1:**
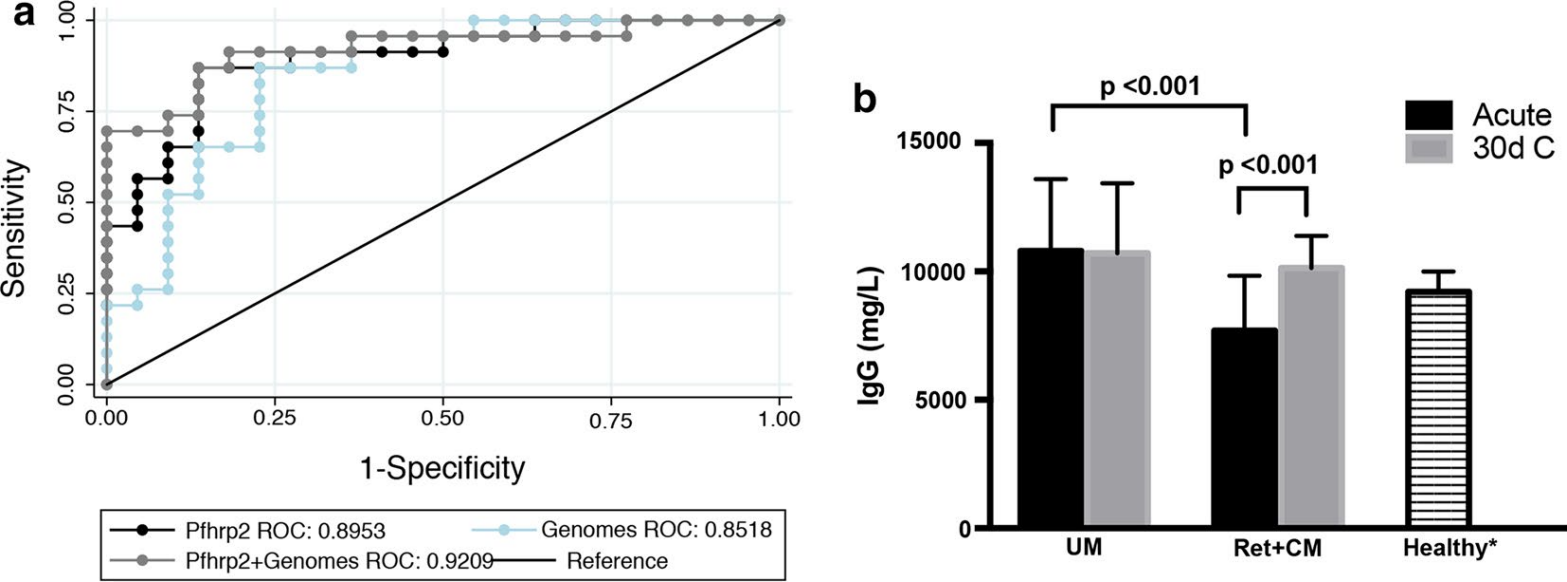
Children with Ret + CM have greater parasite burden and suppressed total IgG in acute infection. **a** Receiver operator characteristics (ROC) curves generated from logistic regression models (PfHRP2, parasite genomes, PfHRP2 + parasite genomes) show that PfHRP2 and cell-free parasite genomes (cfPf DNA) differentiate children by case severity. Cell free parasite DNA is represented as *P. falciparum* genomes from cell free DNA qPCR, and PfHRP2 indicates plasma PfHRP2. **b** Median values of total IgG (mg/L) in acute infection and convalescence as measured by RID reveal suppression of total IgG in acute CM. P-values correspond to Wilcoxon’s rank-sum (unpaired) or signed rank (paired) tests. Healthy* data refers to sub-Saharan African children as described by Obiandu et al. [40]

A total of 25 children with Ret + CM and 23 with UM were included in the study (Table 1). Both groups had similar age and sex distributions. Children with Ret + CM had significantly elevated levels of PfHRP2 [37] and cell-free plasma parasite DNA (‘cfPf DNA’) [30] (both P < 0.001), two biomarkers of parasite burden. PfHRP2 predicts Ret + CM and progression to severe disease [29, 38], and the combination of PfHRP2 and cell-free DNA was the best correlate of severe disease [39] (Fig. 1a). Children with Ret + CM had lower hemoglobin levels (Median: 7.4 vs. 9.6 g/dL, P < 0.001) and lower platelet counts (P = 0.03).

### Children with CM were exposed to a more diverse/parasite population during acute infection than children with UM

As reported previously [25], the PfEMP1 peripheral blood transcript profile data was available for all paediatric malaria cases described here. Children with Ret + CM were exposed to greater parasite diversity (more variant domains) than children with UM (Median number of domains amplified: 21 vs. 11, P < 0.001) (Table 1).

### Total IgG is reduced in acute Ret + CM, but not in UM, and normalizes by 30 days post infection

Ret + CM cases had significantly reduced levels of total IgG relative to children with UM during acute infection (P < 0.001), which normalized in convalescence (Fig. 1b). UM IgG levels were similar to healthy sub-Saharan African children [40] and did not differ across time points. At both time points, total IgG levels correlated significantly with age (acute, rho = 0.50, P < 0.001; convalescent, rho = 0.47, P < 0.001) (see Additional file 4A).

### Children with Ret + CM and UM have similar malaria-specific antibody profiles

**Fig. 2:**
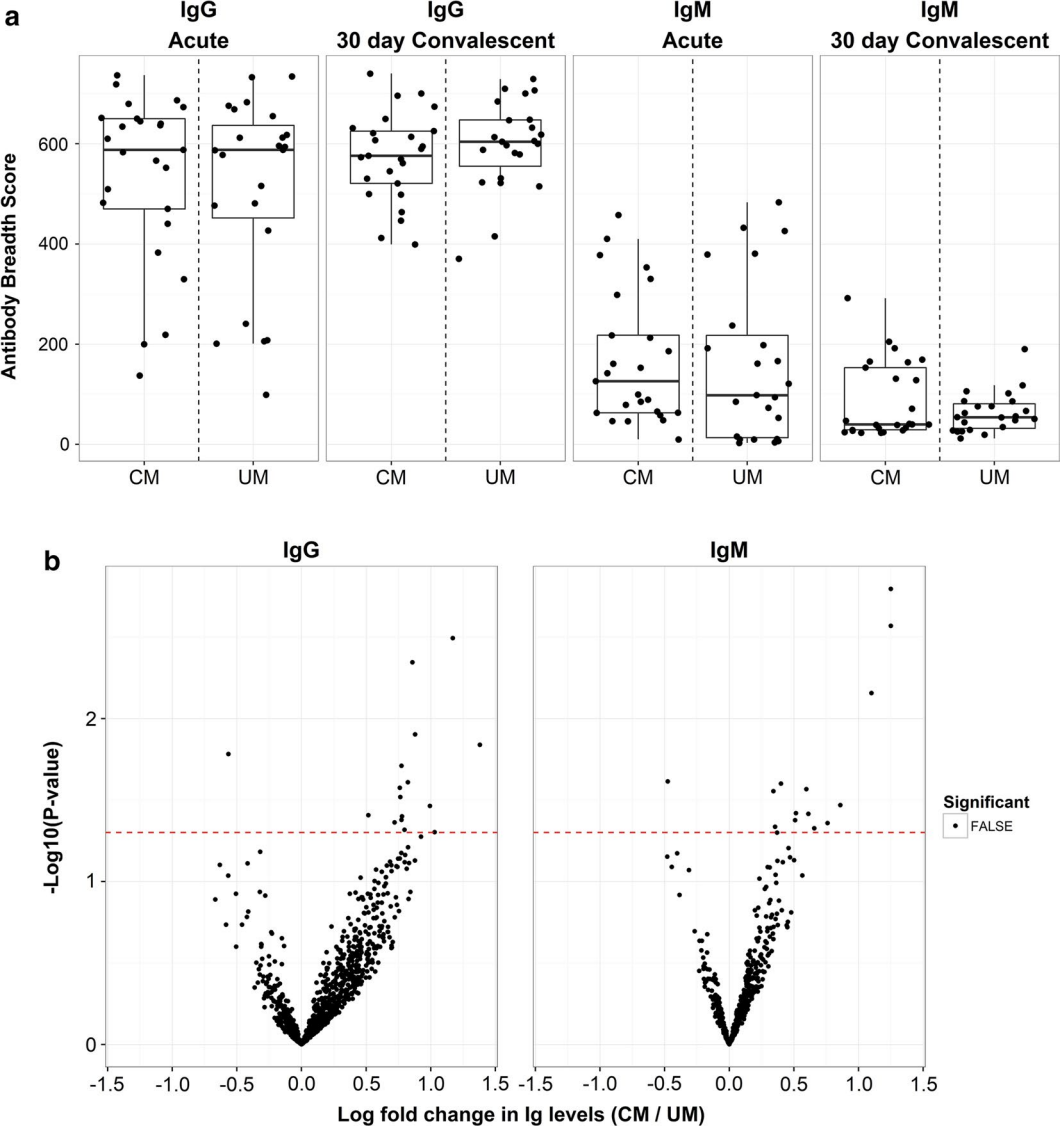
Breadth and magnitude of *Plasmodium falciparum*-specific seroreactivity in Ret + CM cases parallels UM cases. **a** *Plasmodium falciparum* antibody breadth scores (number of seropositive responses) for each case are displayed in boxplots with overlaid data points by timepoint, case severity, and Ig type. Broad responses to *P. falciparum* antigens are detected across timepoints with similar seroreactivity between CM and UM cases. **b** Volcano plots display inverse unadjusted P-values (y-axis) comparing magnitude of IgG and IgM seroreactivity (x-axis) to *P. falciparum* antigens in acute infection by case severity (> 0 indicating higher in Ret + CM cases). Dashed red line represents unadjusted P-value of 0.05. Points above the line have significant unadjusted P-values, but none are significant after adjustment for the false discovery rate (Benjamini-Hochberg). The magnitude of *P. falciparum* reactivity is not significantly different between Ret + CM and UM cases, although there is a trend toward greater reactivity in CM cases

Both Ret + CM and UM cases had a broad repertoire of *P. falciparum*-specific antibody during acute infection (Fig. 2a) with no significant differences in the magnitude of IgG reactivity to any of the *P. falciparum* antigens (Fig. 2b). There were no significant differences in antibody breadth (Fig. 2a) by case severity in convalescence. Age and sex had minimal impact on *P. falciparum*-specific antibody levels (see Additional file 4B, C). It should be noted that the children in this study were younger (Table 1) than most studies that have examined *P. falciparum*-specific antibody profiles of cohorts of symptomatic and asymptomatic children. Children with mild symptomatic malaria were compared to children with clinical CM, a syndrome most common in children under 5 years of age.

Helb et al. reported antigens predictive of recent malaria exposure in Ugandan children using protein microarray [41]. Eight of the 10 antigens included in their exposure model were present on the Pf1000 microarray. For six of the eight antigens, high seroprevalence and reactivity in acute infection with a significant increase in reactivity at 30 days of convalescence was observed (Table 2; For controls see Additional file 5).

**Table 2:** Reactivity to markers of prior malaria exposure and antigens of interest for all cases (UM and Ret + CM n = 48)

Antigen/marker	Gene ID	Seroprevalence	Acute R	Convalescent R	P-value*	
Plasmodium exported protein (GEXP18)	PF3D7_0402400	98 (47)	4.22 (3.69–4.74)	4.65 (4.28–5.02)	*0.01*	
Exonuclease, putative	PF3D7_1106300	85 (41)	2.30 (1.85–2.76)	2.83 (2.39–3.27)	*0.02*	Markers of prior exposure
Erythrocyte membrane protein 1 (VAR)	PF3D7_0711700	100 (48)	4.51 (4.11–4.91)	4.73 (4.38–5.07)	0.13	
Erythrocyte membrane protein 1 (VAR)	PF3D7_0800300	100 (48)	4.29 (3.83–4.74)	4.57 (4.17–4.97)	0.08	
Heat shock protein 40, type II (HSP40)	PF3D7_0501100	100 (48)	3.29 (2.77–3.80)	4.58 (4.24–4.93)	*<* *0.0001*	Helb et al. *PNAS*, [41]
Early transcribed membrane protein 4 (ETRAMP4)	PF3D7_0423700	100 (48)	3.71 (3.23–4.19)	4.18 (3.83–4.53)	*0.02*	
Acyl-coA synthetase (ACS5)	PF3D7_0731600	79 (38)	2.36 (1.83–2.88)	2.85 (2.30–3.41)	*0.01*	
PF70 protein (PF70)	PF3D7_1002100	100 (48)	5.12 (4.77–5.47)	5.69 (5.45–5.93)	*0.01*	
Circumsporozoite protein (CSP)	PF3D7_0304600	44 (21)	0.95 (0.72–1.18)	0.99 (0.79–1.18)	0.67	Vaccine candidates and antigens of interest
Apical membrane antigen 1 (AMA1)	PF3D7_1133400	98 (47)	3.31 (2.83–3.79)	3.60 (3.27–3.94)	0.12	
Merozoite surface protein 1 (MSP1)	PF3D7_0930300	100 (48)	4.51 (3.97–5.05)	5.04 (4.67–5.40)	*0.02*	
Merozoite surface protein 2 (MSP2)	PF3D7_0206800	98 (47)	4.01 (3.51–4.50)	4.29 (3.88–4.69)	0.12	
Erythrocyte binding antigen-175 (EBA175)	PF3D7_0731500	92 (44)	2.40 (1.96–2.84)	3.03 (2.64–3.41)	*0.003*	
Glutamate-rich protein (GLURP)	PF3D7_1035300	88 (42)	2.47 (1.98–2.96)	2.53 (2.06–3.00)	0.70	
Liver stage antigen 1 (LSA1)	PF3D7_1036400	100 (48)	3.70 (3.28–4.12)	4.00 (3.68–4.33)	0.08	
Liver stage antigen 3 (LSA3)	PF3D7_0220000	100 (48)	4.45 (4.01–4.90)	5.50 (5.26–5.73)	*<* *0.0001*	

Reactivity (R), mean (95% CI); * P-values correspond to Benjamini–Hochberg adjusted paired empirical Bayes t-test, Acute vs. Convalescent

Seroprevalence, % (n) of cases with positive seroreactivity. R, seroreactivity where R >= 1 indicates a seropositive response

Italic values indicate P < 0.05

Reactivity to *P. falciparum* vaccine candidates and candidate protective antigens was also examined [5, 42]. In acute samples, high seroprevalence and reactivity was observed with significantly greater reactivity at 30 days of convalescence for MSP1, EBA175, and LSA3 (P < 0.05, < 0.01, < 0.01, respectively) (Table 2). Seroprevalence to CSP, the target of the RTS, S vaccine, was just below 50%, with moderate reactivity. When seroreactivity to antigens previously identified as potential correlates of protection from symptomatic disease using protein microarrays [5] was compared, no differences were observed by case severity.

### PfEMP1 (± ATS) specific reactivity in acute infection is similar between UM and CM cases

Next, the relationship/correlation between PfEMP1 antibody reactivity in acute infection and differences in parasite burden or clinical status was assessed. The ATS portion of each PfEMP1 was expressed on a different spot on the array than the extracellular domains, and each 3D7 PfEMP1 was represented by 2 or 3 different spots that encompassed non-overlapping segments of the coding region. Unlike extracellular domains, the intracellular acidic terminal sequence (ATS) portion of each PfEMP1 protein is highly conserved. Therefore, all analyses examining antibody reactivity to PfEMP1 proteins were performed with and without the ATS.

**Fig. 3:**
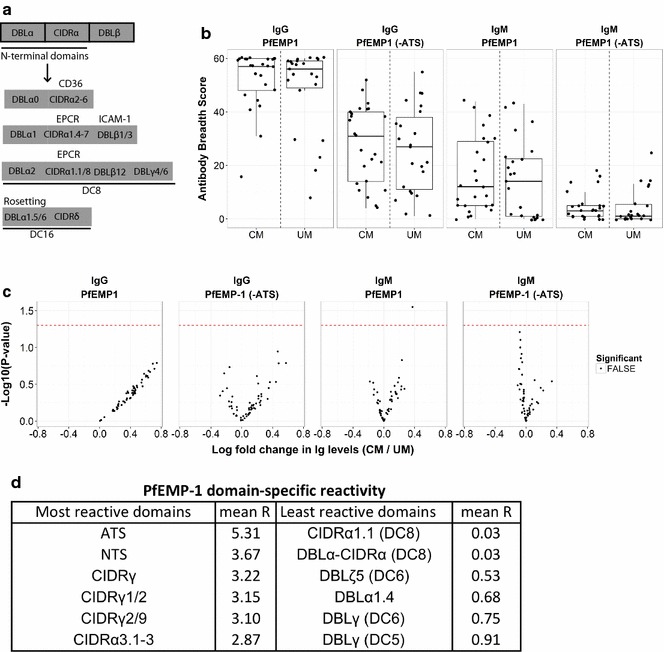
Acute paediatric malaria cases have broad reactivity to PfEMP1 antigens with lowest reactivity to EPCR-binding CIDR domains. **a** Schematic of PfEMP1 domain structure. **b** PfEMP1 breadth scores are displayed in boxplots with overlaid data points by case severity and Ig type in acute infection. There were broad responses and similar seroreactivity despite differences in disease severity. **c** Volcano plots display inverse unadjusted P-values (y-axis) comparing magnitude of IgG, IgM seroreactivity (x-axis) to PfEMP1 antigens in acute infection by case severity (to the right of 0 indicates higher in Ret + CM cases). Data suggest magnitude of total PfEMP1 reactivity is not different between Ret + CM and UM. Red dashed lines represent unadjusted P-value of 0.05. Points above the line have significant unadjusted P-values, but none are significant after adjustment for the false discovery rate (Benjamini-Hochberg). **d** Most and least seroreactive PfEMP1 antigens during acute infection. The most (top 6) and least (bottom 6) reactive PfEMP1 antigens are indicated (see Additional file 2 for details). Reactivity of all UM and CM cases was scored and maximal reactivity during acute infection was used to generate a mean reactivity (R) value. Conserved domains (e.g. ATS) were the most reactive

By case severity, no significant differences in the breadth and magnitude of IgG and IgM responses to PfEMP1 specific antigens were observed in acute infection (Figs. 3b, c). After excluding reactivity to the ATS, the median breadth score to PfEMP1 antigens in acute infection for both Ret + CM and UM cases was ~ 30/60 (Fig. 3b).

### All children (UM and Ret + CM) seroreact to PfEMP1 domains with apparent high reactivity to ‘non-virulent’ PfEMP1 domains and low reactivity to ‘virulent’ domains in acute infection

As no significant differences in reactivity by case severity (UM *vs.* Ret + CM) were observed, all cases were pooled for subsequent PfEMP1 specific analyses. PfEMP1 antigens with the highest reactivity in acute infection were highly conserved ATS regions or domains associated with CD36 binding (Fig. 3d). The ATS region is intracellular, and reactivity to this conserved region is a measure of exposure and not protection. The least reactive antigens during acute infection were DC8 and group A antigens associated with EPCR-binding or rosetting phenotypes, both associated with severe disease in paediatric malaria (Fig. 3d). Reactivity across all binding groups was observed in acute infection but to a lesser extent than observed in control sera pooled from hyperimmune (Malawian) IgG (see Additional file 6).

### Children exposed to specific PfEMP1 during acute infection do not mount specific IgG responses in convalescence that are detectable by protein microarray

**Fig. 4:**
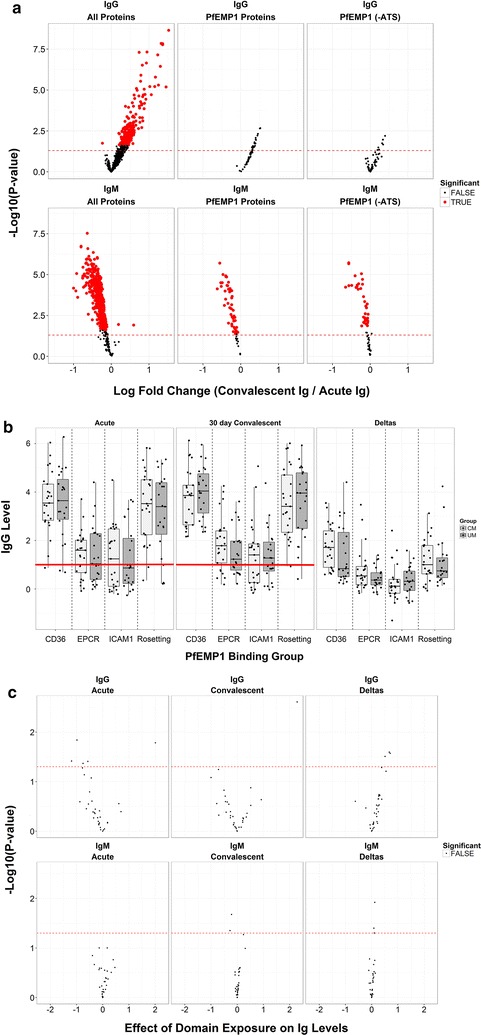
PfEMP1 (domain) exposure in acute infection does not affect seroreactivity to corresponding antigen(s) in convalescence. **a** Volcano plots displaying inverse unadjusted P-values (y-axis) comparing the change in IgG, IgM seroreactivity to *P. falciparum* and PfEMP1 antigens in convalescence (x-axis; points to the right of 0 indicate greater reactivity in convalescence). While IgM declines in a classical fashion in convalescence for both total and PfEMP1 specific antigens, IgG responses to PfEMP1 antigens are not mounted in convalescence. Red dashed lines represent unadjusted P-value of 0.05. Points above the line have significant unadjusted P values, but only red bold points are significant after adjustment for the false discovery rate. **b** Magnitude of responses to PfEMP1 antigens is displayed in boxplots with overlaid data points by CIDR-specific binding group for Ret + CM cases (dark grey) and uncomplicated malaria (light grey). Seroreactivity in acute infection is lowest to EPCR-binding domains and ICAM-1 binding domains linked to EPCR. Children with exposure to these antigens as determined by qRT-PCR do not mount responses to these antigens in convalescence. Red lines indicate seroreactivity (R) = 1, where R > 1 indicates a seropositive response. **c** Volcano plot of linear regression effect estimates (x-axis) for the independent variable of exposure (Y/N) in the exposure models generated for PfEMP1 domains amplified by qRT-PCR from acute samples indicates that exposure in acute infection does not correlate with seroreactivity to the corresponding antigen in convalescence

Upon comparing longitudinal changes in Ig reactivity to all Pf and PfEMP1-specific antigens, both overall Pf and PfEMP1 (± ATS) specific IgM responses decline significantly in convalescence for all cases (Fig. 4a). While overall Pf IgG responses increase significantly in convalescence, PfEMP1 (± ATS) specific IgG responses do not (Fig. 4a).

When the PfEMP1 antigens were classified on the basis of their known or predicted binding phenotype [15, 43], 5 EPCR-binding antigens, 2 rosetting antigens, 45 predicted CD36 binding antigens were represented on the array. Additional file 7 shows total responders and responders by case severity during acute infection. The highest breadth scores were observed for rosetting antigens. UM and Ret + CM children had a lower magnitude of response to EPCR-binding antigens than to either CD36-binding or rosetting antigens (Fig. 4b). A Group A ICAM-1 binding motif is often linked to EPCR binding [32], and there was a similar magnitude of antibody response to these domains (Fig. 4b) between groups despite a significant difference in exposure to the corresponding antigen/motif during acute infection (Percent exposed: 52.6% vs. 21.7%, P = 0.04) (Table 1). The total number of responders (seroprevalence) to each CIDR antigen was variable but not significantly different across binding phenotype group (Additional file 6). Of note, seroprevalence to the Group A-ICAM-1 binding domain was greater than to linked CIDRα1 domains suggesting greater cross reactivity of antibodies to ICAM-1-binding domains (Table 3 and Additional file 8).

**Table 3:** Reactivity to head structure/CIDR or ICAM-1 binding domain in exposed cases

Binding phenotype	Pf1000 array gene ID	Binding domain	Exposed (n of 48)	Acute responders, % (n)	New responders, % (n)	Non responders, % (n)
EPCR						
	PF3D7_0400400	CIDRα1.1	26	4% (1)	4% (1)	92% (24)
	PF3D7_1150400	CIDRα1.4^b^	2	0% (0)	0% (0)	100% (2)
	PF3D7_0800300	CIDRα1.6	9	22% (2)	22% (2)	56% (5)
	PF3D7_0425800	CIDRα1.6^b^	9	11% (1)	0% (0)	89% (8)
ICAM-1^a^						
	PF3D7_1150400	DBLβ3	25	28% (7)	4% (1)	68% (17)
	PF3D7_0425800	DBLβ3	25	36% (9)	4% (1)	60% (15)
Rosetting						
	PF3D7_1300300	CIDRδ1	12	67% (8)	8% (1)	25% (3)
	PF3D7_0800200	CIDRδ2	12	0% (0)	25% (3)	75% (9)
CD36						
	PF3D7_1200100	CIDRα2.2	8	50% (4)	12% (1)	38% (3)
	PF3D7_0200100	CIDRα2.2	8	0% (0)	0% (0)	100% (8)
	PF3D7_0809100	CIDRα2.2	8	50% (4)	0% (0)	50% (4)
	PF3D7_0200100	CIDRα2.2	8	88% (7)	12% (1)	0% (0)
	PF3D7_1255200	CIDRα2.3	32	12% (4)	6% (2)	82% (26)
	PF3D7_1041300	CIDRα2.7	32	72% (23)	12% (4)	16% (5)
	PF3D7_0808700	CIDRα3.1	14	50% (7)	29% (4)	21% (3)
	PF3D7_1000100	CIDRα3.1	14	50% (7)	14% (2)	36% (5)
	PF3D7_0712900	CIDRα3.1	14	0% (0)	7% (1)	93% (13)
	PF3D7_1240600	CIDRα3.1	14	7% (1)	0% (0)	93% (13)
	PF3D7_0937800	CIDRα3.1	14	64% (9)	7% (1)	29% (4)
	PF3D7_0412900	CIDRα3.1	14	7% (1)	0% (0)	93% (13)
	PF3D7_0712600	CIDRα3.1	14	36% (5)	0% (0)	64% (9)
	PF3D7_0712000	CIDRα3.1	14	0% (0)	7% (1)	93% (13)
	PF3D7_0833500	CIDRα3.1	14	43% (6)	14% (2)	43% (6)
	PF3D7_0632500	CIDRα3.2	14	64% (9)	21% (3)	14% (2)
	PF3D7_0420700	CIDRα3.2	14	0% (0)	7% (1)	93% (13)
	PF3D7_0420900	CIDRα3.2	14	0% (0)	0% (0)	100% (14)
	PF3D7_0711700	CIDRα3.2	14	0% (0)	0% (0)	100% (14)
	PF3D7_0412700	CIDRα3.2	14	14% (2)	7% (1)	79% (11)
	PF3D7_0808600	CIDRα3.2	14	14% (2)	7% (1)	79% (11)
	PF3D7_1100100	CIDRα3.2	14	29% (4)	21% (3)	50% (7)
	PF3D7_0421300	CIDRα3.4	26	15% (4)	0% (0)	85% (22)
	PF3D7_0900100	CIDRα3.4	26	35% (9)	15% (4)	50% (13)
	PF3D7_1219300	CIDRα3.4	26	15% (4)	8% (2)	77% (20)
	PF3D7_1373500	CIDRα3.4	26	11% (3)	4% (1)	85% (22)
	PF3D7_0733000	CIDRα3.4	26	0% (0)	4% (1)	96% (25)
	PF3D7_0223500	CIDRα3.4	26	50% (13)	15% (4)	35% (9)
	PF3D7_1240400	CIDRα3.4	26	50% (13)	8% (2)	42% (11)

Exposed, domain amplified in acute infection

Responders: Acute, reactive in acute infection; New, reactive only in convalescence; Non, not reactive

^a^Exposure status available for n = 42

^b^Head structure/CIDR domains followed by ICAM-1 DBLβ3 domain (CIDRα1-DBLβ)

Exposure to PfEMP1 domains during acute infection was determined using relative qRT-PCR profiling of *var* transcripts in the peripheral blood of each case (both UM and Ret + CM) as previously described [19, 25, 32, 44]. As no significant differences in seroreactivity to Pf1000 antigens by case severity (UM vs. Ret + CM) was detected, PfEMP1 exposed cases were assessed altogether for antibody reactivity to PfEMP1 expressed during acute infection focusing upon CIDR domains across three major binding phenotypes. A substantial antibody response at follow-up in children exposed to a given PfEMP1 antigen was not detected (Table 3; see unexposed individuals in Additional file 8). A single exposure model was generated for each PfEMP1 domain with PfEMP1 expression data available. Exposure to a PfEMP1 antigen in acute infection had no correlation with the antibody level to that antigen in convalescence (Fig. 4c). Age and sex were the only variables that significantly affected convalescent (or delta) antibody levels, and both variables affected IgM but not IgG levels (see Additional file 9). Control adult immune IgG seroreactivity was greater than that observed in children (see Additional files 3, 5).

## Discussion

Differences in antibody repertoire between UM and CM cases could explain why some children with *P. falciparum* infection progress to severe disease. Despite evidence of general suppression of antibody response in acute Ret + CM (Fig. 1), similar robust reactivity to a broad range of *P. falciparum* antigens was seen in UM and CM cases alike (Fig. 2). Reactivity to unfractionated malaria antigen and specific antigens of interest (e.g. MSP-1, CSP) by EIA or ELISA in children from the Gambia showed that evidence of prior exposure did not prevent progression to severe disease [45]. Here, a large proportion of the *P. falciparum* proteome was assayed and no differences in the breadth or magnitude of *P. falciparum* reactivity by case severity were found (UM vs. Ret + CM; Fig. 2). The antigens on the array were selected based upon their interest as vaccine or diagnostic candidates as well as seroreactivity in both natural and experimental infection and enabled measurement of antibody responses during acute disease and in convalescence. While it cannot be definitively ascertained whether antibody present during acute disease reflects current infection or prior infection, a similar pattern in IgM reactivity that is classically indicative of acute infection was observed in the UM and CM groups across both time points, suggesting that the level of prior exposure and generation of antibody to *P. falciparum* antigens in Ret + CM cases parallels that of children with UM.

In a study employing an 824 feature *P. falciparum* protein microarray, antibody to a subset of antigens (including MSP1, MSP2, LSA1, LSA3, Pf70, and PfEMP1; Table 2) was associated with protection from symptomatic disease in sub-Saharan African children [5]. Many of these antigens are current or previous vaccine candidates [42, 46–48] and have inconsistently been reported as protective in the literature [4, 7, 49, 50]. Independent of disease severity, high seroreactivity to this group of antigens was identified in children with symptomatic malaria (Table 2). Thus, high seroreactivity is common across children living in endemic regions, such as Malawi, and may be a marker of prior (or repeated) *P. falciparum* exposure. Dent et al. followed a cohort longitudinally, monitoring seroreactivity pre- and post- infection [5], as did Crompton et al. [12]. Longitudinal studies suggest that the antibody response of younger children to malaria antigens including PfEMP1 is short-lived [12, 26]. The antibody response to *P. falciparum* appears to increase with age, and it is not yet clear what factors promote antibody stability and which antibodies are important for the prevention of symptomatic disease versus for prevention of progression to severe manifestations such as CM. Taken together with the results presented here, the data suggests that antibody against some of these targets may be more important for the prevention of symptomatic disease than for the progression to severe manifestations, such as CM.

With regard to PfEMP1 specific antigens, and in agreement with previous studies [5] [27], magnitude of reactivity to the highly conserved ATS proteins was greatest. As the ATS is an intracellular domain of PfEMP1, reactivity to this region should be regarded as a surrogate of exposure rather than protection. The CIDR domains are the most hypervariant of all PfEMP1 domains/regions, and seroreactivity to CIDR domains in the patient population described here was highly variable across sequence type (i.e. α/β/γ) and subtype (e.g. CIDRα1) (Fig. 3 and Additional file 7). Within the subset of CIDR domains, reactivity to PfEMP1 domains associated with EPCR-binding was lowest and reactivity to those predicted to bind CD36 was highest [43]. The breadth and magnitude of response to all EPCR-binding CIDR domains on the microarray was lower in acute infection than that to CD36-binding or rosetting CIDR domains (Fig. 3d). It is important to note that while the strongest seroreactivity signal observed corresponded to CD36-binding CIDR domains, there are more probes that encode this binding property on the array (45 vs. 5 EPCR-binding and 2 rosetting) yielding a higher likelihood of measuring a maximum response as a central measure of IgG reactivity. Abundance of features does not fully explain our results, as there are over twice as many EPCR-binding antigens on the array as rosetting antigens, yet the seroreactivity to EPCR-binding antigens remains lowest. The PfEMP1 antigens on the Pf1000 array that were annotated for this study contain two adjacent domains: DBL-CIDR head structure (N-terminus) domains or in the case of ICAM-1 binding PfEMP1 antigens, DBL–DBL domains (see Additional file 3). Further studies of the specificity of seroreactivity observed for individual binding domains or to variants from other sequenced strains can be pursued readily using the same platform, and the consistent annotation schema provide an initial view of antibody responses to different classes of PfEMP1.

Seroreactivity to the newly identified *var A* ICAM-1 binding motif (2 spots on the array) [32] was found to parallel seroreactivity to EPCR-binding head structures (Fig. 4b). EPCR-binding PfEMP1 often also encode ICAM-binding motifs (Table 3 and Additional file 7). While the magnitude of seroreactivity to this ICAM-1 binding motif was lower than that to CD36-binding and rosetting PfEMP1, a greater fraction of children had detectable antibodies to this motif compared with PfEMP1 extracellular domains associated with EPCR-binding. Exposed children and adults in malaria endemic regions have reactive, inhibitory IgG to this conserved ICAM-1 binding motif, possibly reflecting the greater conservation of the ICAM-1 binding motif than the EPCR-binding CIDR [32]. Here, the data demonstrating greater seroprevalence in children to this motif than to linked CIDR domains is consistent with those results. In adult hyperimmune sera, seroreactivity to ICAM-1 domains on the array was also greater than seroreactivity to EPCR domains (see Additional file 6).

Neither a consistent response to the corresponding antigen in children exposed to a specific PfEMP1 variant in acute infection or a general significant increase in PfEMP1 IgG antibody at 30 days convalescence were detected. Prior studies employing in vitro functional assays demonstrated that children seroconvert against the VSA of the isolate seen in acute infection following disease resolution [51] and subsequent studies suggest that the major VSA recognized by immune sera is PfEMP1 [52]. An array study performed in Papua New Guinea showed that the anti-PfEMP1 antibody response was more pronounced in children with detectable parasitaemia [26]. It is possible that the study subjects made PfEMP1-specific antibody in response to acute infection, but this antibody was not maintained in convalescence.

The array was developed using the 3D7 proteome, which lacks some EPCR-binding proteins. 3D7 PfEMP1 proteins may lack sufficient homology to Malawian PfEMP1 to be recognized by specific antibody. The PfEMP1 family is hypervariable with less than 50% sequence identity found in homologous domains of homologous genes [15, 16]. Strain-specific polymorphism of MSP1 and AMA1, vaccine antigens that are more conserved than PfEMP1, is proposed to be a major limiting factor in development of protective antibodies [53]. Additionally, incomplete or inaccurate protein folding for certain PfEMP1 antigens on the array may also contribute to the observed differences across studies, especially since many *P. falciparum*-specific antibodies recognize conformational epitopes. The cell free in vitro expression system and direct printing protocol used in production of the microarray employed here involves minimal manipulation of expressed proteins and no denaturation steps, but correct conformation of recombinant protein is not readily verifiable.

Seroreactivity in both acute infection and convalescence to antigens from all PfEMP1 binding phenotype groups was identified in UM and CM cases. Seroreactivity to EPCR domains is acquired relatively early in life [28] and may explain why older children are less likely to develop CM. Children with symptomatic malaria had a lower level of seroreactivity to EPCR-binding *P. falciparum* antigens than to other PfEMP1 antigens. The median age of our subjects was 3–4 years, whereas peak EPCR-binding domain antibody prevalence was detected in children aged 8–10 years [28]. Although the rate of antibody acquisition is dependent upon a number of factors including exposure to *Plasmodium,* studies that have examined acquisition of “protective” antibody responses to VSA or *Plasmodium* antigens, significant protection begins to appear in children 8–10 years of age [12, 52]. Children appear to be at risk for symptomatic malaria until they are older than the children described here. Pooled immune IgG from Malawian adults [34] (see Additional file 6) was seroreactive across all PfEMP1 binding groups. Multiple exposures are likely required to develop significant cross-reactive antibody to hypervariable extracellular PfEMP1 domains. An array that encompasses Malawian PfEMP1 sequences, as reported for the Papua New Guinea study [26], may be useful to determine when children with malaria develop antibodies to local PfEMP1 variants.

Antibody reactivity to PfEMP1 binding groups should be compared with caution and the findings would be strengthened by supplemental functional assays and comparison with other platforms [25]. The high throughput microarray provides a cost-effective platform to evaluate reactivity to a large number of *P. falciparum* antigens using small volumes of sample and is readily amenable to simultaneous querying of reactivity of sera to proteins from strains of disparate genetic backgrounds, as recently demonstrated for AMA1 and MSP1 [53]. Due to recent advances in HIV vaccine efforts, there has been renewed interest in vaccine development of a number of infectious diseases associated with chronic antigen exposure. For many of these diseases, including malaria, there is often a robust host antibody response but very slow acquisition of disease-modifying immunity. Given progress in HIV vaccine research, there has been renewed interest in identifying strain-transcendent protective epitopes.

## Conclusion

Despite differences in clinical presentation and parasite burden, no significant differences in breadth or magnitude of *P. falciparum* seroreactivity between children with Ret + CM and children with UM were found using a 1000 feature *P. falciparum* protein microarray. This suggests that all children had similar levels of prior exposure and supports the notion that children experience uncomplicated malaria episodes in between severe malaria episodes [54]. Children presenting with acute malaria have significant breadth and magnitude of *P. falciparum* antibody, indicating these antibodies do not preclude progression to symptomatic malaria or more severe disease. Conserved domains of PfEMP1 are more prominent targets of cross reactive antibodies than variable domains in children with symptomatic malaria. The key determinants required for formation of protective antibodies and surrogates of antibody protection require further investigation. Studies of *P. falciparum*-specific antibody repertoires that incorporate a broader range of strain-specific PfEMP1 antigens with functional assays to characterize protective antibody function will improve the currently incomplete understanding of the role of antibody immunity in prevention of symptomatic paediatric malaria.

## Additional files

**Additional file 1.** Consort diagram of patient recruitment and study design. All cases were recruited from QECH in Blantyre, Malawi. Cases were included in the study if they attended their assigned 30-day follow-up appointment and fit the comparable age and sex distributions (for adjusted comparison) between groups.

**Additional file 2.** Supplemental methods for array design, probing, and analysis.

**Additional file 3.** Annotations of PfEMP1/*var* genes and domains encoded.

**Additional file 4.** Assessment of *P. falciparum* and total Ig levels by age and sex. (a) Spearman correlations comparing age vs. acute total IgG level, age vs. 30-day convalescent total IgG level, and total IgG levels across timepoints show significant linear trends. (b) Volcano plots comparing the effect of age (> 5 years/≤ 5 years) on IgG, IgM seroreactivity to *P. falciparum* and PfEMP1 antigens indicates that for IgG in acute infection, age affects the magnitude of seroreactivity to a few *P. falciparum* antigens and a general non-significant trend for higher antibodies to all proteins in older children during acute infection (CM + UM total n = 48). (c) Volcano plots of inverse unadjusted P-values (y-axis) and linear regression effect estimates (x-axis) for comparing the effect of sex (male/female) on IgG, IgM seroreactivity to *P. falciparum* and PfEMP1 antigens indicate that sex does not have a significant effect on seroreactivity in our patient population in acute infection or convalescence, although non-significant trends for higher overall *P. falciparum*-specific IgG and IgM in males during acute infection and lower PfEMP1-specific IgG in males for both time points could be observed (CM + UM total n = 48). Dashed lines represent unadjusted P-value of 0.05. Points are highlighted in bold, red if significant after adjustment for the false discovery rate.

**Additional file 5.** Reactivity to markers of prior malaria exposure and antigens of interest in control populations.

**Additional file 6.** Seroreactivity to PfEMP1 antigens in pooled control serum vs. paediatric malaria cases.

**Additional file 7.** CIDR IgG responders at time of acute *P. falciparum* infection.

**Additional file 8.** Reactivity to head structure/CIDR or ICAM-binding domain in unexposed cases.

**Additional file 9.** Additional exposure model covariates: the effect of cerebral malaria, age, and sex on PfEMP1 antibody level. Volcano plots of linear regression effect estimates (x-axis) for the independent variables (cerebral malaria, Ret + CM/UM; age, ≤ 5/> 5; sex, male/female) included in the exposure models generated for PfEMP1 expressed domains as determined by qRT-PCR of infected blood samples obtained during acute disease. (Total n = 48; CM n = 25; > 5 years of age n = 14; male n = 32).
