# Supplementary material for: Convalescent Plasmodium falciparum-specific seroreactivity does not correlate with paediatric malaria severity or Plasmodium antigen exposure
Source: Malar J. 2018 Apr 25;17:178. doi: 10.1186/s12936-018-2323-4 (PMC5918990; doi:10.1186/s12936-018-2323-4)
Supplement: Supplementary file 1 — Additional file 1. Consort diagram of patient recruitment and study design. All cases were recruited from QECH in Blantyre, Malawi. Cases were included in the study if they attended their assigned 30-day follow-up appointment and fit the comparable age and sex distributions (for adjusted comparison) between groups. [file 12936_2018_2323_MOESM1_ESM.pdf]

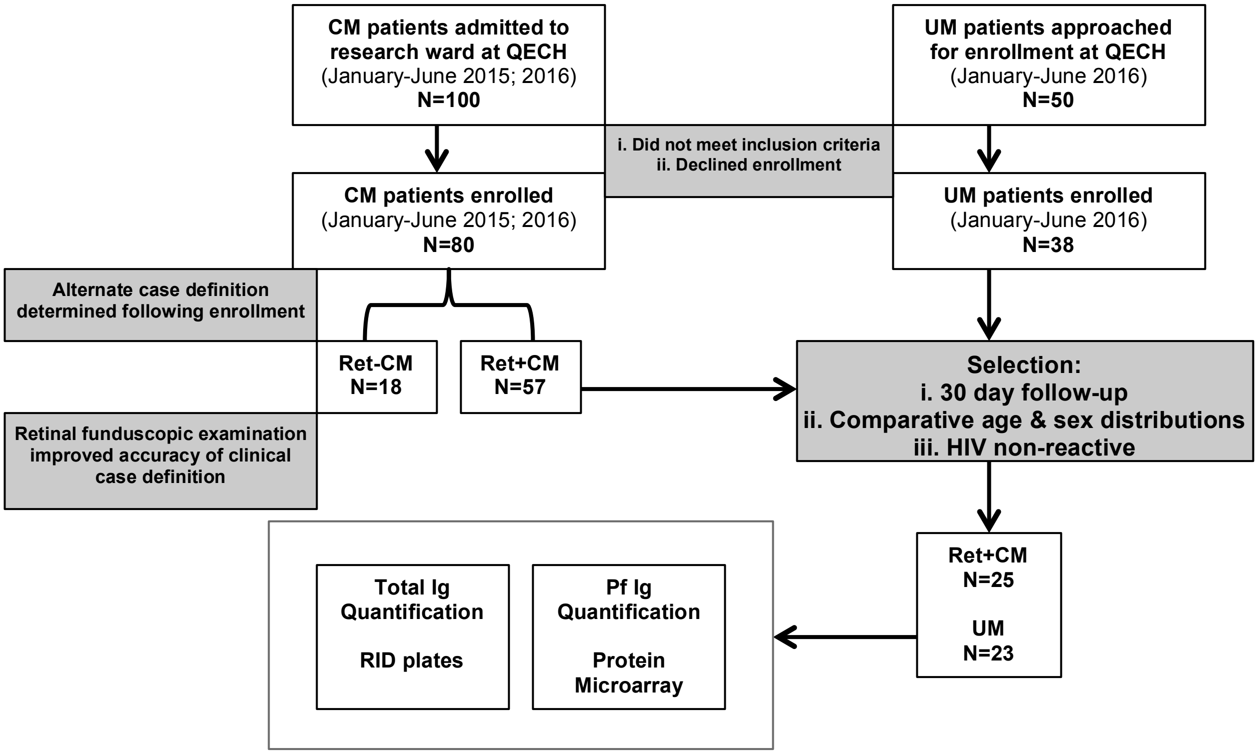
CM patients admitted to research ward at QECH
(January-June 2015; 2016)
N=100
UM patients approached for enrollment at QECH
(January-June 2016)
N=50
i. Did not meet inclusion criteria
ii. Declined enrollment
CM patients enrolled
(January-June 2015; 2016)
N=80
UM patients enrolled
(January-June 2016)
N=38
Alternate case definition determined following enrollment
Ret-CM
N=18
Ret+CM
N=57
Selection:
i. 30 day follow-up
ii. Comparative age & sex distributions
iii. HIV non-reactive
Retinal funduscopic examination improved accuracy of clinical case definition
Ret+CM
N=25
UM
N=23
Total Ig Quantification
RID plates
Pf Ig Quantification
Protein Microarray
