# Supplementary material for: Convalescent Plasmodium falciparum-specific seroreactivity does not correlate with paediatric malaria severity or Plasmodium antigen exposure
Source: Malar J. 2018 Apr 25;17:178. doi: 10.1186/s12936-018-2323-4 (PMC5918990; doi:10.1186/s12936-018-2323-4)
Supplement: Supplementary file 2 — Additional file 2. Supplemental methods for array design, probing, and analysis. [file 12936_2018_2323_MOESM2_ESM.docx]

**Additional file**

**Methods**

**Protein microarray fabrication.** Proteins were expressed using an *in vitro* transcription and translation (IVTT) system, the *Escherichia coli* cell-free Rapid Translation System (RTS) kit (5 Prime, Gaithersburg, MD, USA). A library of partial or complete open reading frames (ORFs) cloned into a T7 expression vector pXI has been established at Antigen Discovery, Inc. (ADI, Irvine, CA, USA). This library was created through an *in vivo* recombination cloning process with PCR-amplified ORFs, and a complementary linearized expressed vector transformed into chemically competent *E. coli* was amplified by PCR and cloned into pXI vector using a high-throughput PCR recombination cloning method described elsewhere [1]. Each expressed protein includes a 5’ polyhistidine (HIS) epitope and 3’ hemagglutinin (HA) epitope. After expressing the proteins according to manufacturer instructions, translated proteins were printed onto nitrocellulose-coated glass AVID slides (Grace Bio-Labs, Inc., Bend, OR, USA) using an Omni Grid Accent robotic microarray printer (Digilabs, Inc., Marlborough, MA, USA). Each slide contained eight nitrocellulose “pads” for which the full array was printed in replicate, allowing eight samples to be probed per slide. Microarray chip printing and protein expression were quality checked by probing random slides with anti-HIS and anti-HA monoclonal antibodies with fluorescent labeling.

**Protein microarray sample probing.** Prior to sample application, a probing plan was developed to balance plasma samples across microarray slides by the following variables: case severity (UM vs. CM), sex, age, clinical measures during acute infection (temperature, coma score, hemoglobin levels, smear score), additional malaria-associated syndromes (severe anemia, respiratory distress, jaundice), and time of recruitment (month and year). Paired samples (i.e. acute and 30 day FU) from a single patient were probed onto a single chip (8 samples per chip; 4 paired samples (cases) per chip) to allow for accurate time point comparisons.

All slides were selected from a uniform batch produced by a single Accent printer and passed quality control assessments for intra and inter slide consistency prior to experimental use. Briefly, samples were probed at 1:100 (20% *E. coli* lysate; GenScript) overnight at 4° C. Secondary anti-IgG antibody (biotin-SP-conjugated donkey anti-human IgG; Jackson Immuno) was applied the following day at 1:1000 (respectively in 2% *E. coli* lysate; GenScript) for 1 hour at room temperature. Streptavidin Sensilight-P3 (Columbia Biosciences) and rabbit anti-human IgM-Cy3 (Jackson Immuno) were applied to each slide at 1:200 (2% *E. coli* lysate; GenScript) for 1 hour, covered (no light) at room temperature. Washes were performed with TTBS (Tween Tris-buffered saline) 3x before/after all incubations. Prior to overnight drying in a desiccator, all slides were washed with TBS (Tris-buffered saline) 3x and spun dry by centrifugation at 1,000 x g for 4 min. Air dried chips were scanned on a GenePix 4300A High-Resolution Microarray Scanner (Molecular Devices, Sunnyvale, CA, USA), and spot and background intensities were measured using an annotated grid file (.GAL). Data were exported in Microsoft Excel.

**Protein microarray data normalization.** Raw spot and local background fluorescence intensities, spot annotations and sample phenotypes were imported and merged in the R statistical environment (www.r-project.org), where all subsequent procedures were performed. Foreground spot intensities were adjusted by local background by subtraction, and negative values were converted to 1. Next, all foreground values were transformed using the base 2 logarithm. The dataset was normalized to remove systematic effects by subtracting the median signal intensity of the IVTT controls for each sample. Since the IVTT control spots carry the chip, sample and batch-level systematic effects, but also antibody background reactivity to the IVTT system, this procedure normalizes the data and provides a measure of the specific antibody binding relative to the non-specific antibody binding to the IVTT controls (a.k.a. background). With the normalized data, a value of 0.0 means that the intensity is no different than the background, and a value of 1.0 indicates a doubling with respect to background.

**Protein microarray analysis.** Antibody reactivity was determined relative to background signal of the IVTT controls (log_2_  scale), such that target probe signal intensity greater than or equal to 2x the background signal (a normalized value ≥1.0) was considered seropositive. Antibody breadth, represented as breadth scores, was calculated as the sum of seropositive responses per individual. Antibody magnitude (level) was defined as the log_2_-transformed ratio of target signal to background signal, represented by the normalized signal intensity. Antibody “Deltas” were calculated by subtracting normalized antibody levels at 30 day convalescence from acute antibody levels. For PfEMP1 individual proteins, binding groups and cassette domains, where multiple proteins comprise each domain or group, the maximum signal among the corresponding protein spots was used. For example, if one PfEMP1 protein was represented by three protein spots, the maximum value of the three spots was used. In the case of comparisons with and without ATS, the ATS-bearing spot was excluded from calculating of the maximum response. Likewise, if multiple PfEMP1 proteins comprised a domain group or cassette, the maximum response was used for the group. Antibody level comparisons between case group (Ret+CM/UM), sex (M/F), and age (>5/≤5) were tested using empirical Bayes (eBayes) moderated T tests [2], and paired tests were performed for comparisons between acute and 30 day convalescence time points. Antibody breadth was compared using Poisson regression. Effects of exposure (Y/N), case group, sex, and age on antibody levels to individual PfEMP1 domains were estimated using linear regression. Results were displayed in boxplots with overlaid data points and volcano plots, which show the difference or effect estimate between groups (x-axis) by the inverse log_10_ P-value (y-axis). Results were also tabulated for each set of comparisons.

**Supplemental References**

1. Davies DH, Liang X, Hernandez JE, Randall A, Hirst S, Mu Y, Romero KM, Nguyen TT, Kalantari-Dehaghi M, Crotty S *et al*: **Profiling the humoral immune response to infection by using proteome microarrays: high-throughput vaccine and diagnostic antigen discovery**. *Proceedings of the National Academy of Sciences of the United States of America* 2005, **102**(3):547-552.

2. Smyth GK: **Linear models and empirical bayes methods for assessing differential expression in microarray experiments**. *Stat Appl Genet Mol Biol* 2004, **3**:Article3.
