# Supplementary figures and images for: Convalescent Plasmodium falciparum-specific seroreactivity does not correlate with paediatric malaria severity or Plasmodium antigen exposure
Source: Malar J. 2018 Apr 25;17:178. doi: 10.1186/s12936-018-2323-4 (PMC5918990; doi:10.1186/s12936-018-2323-4)

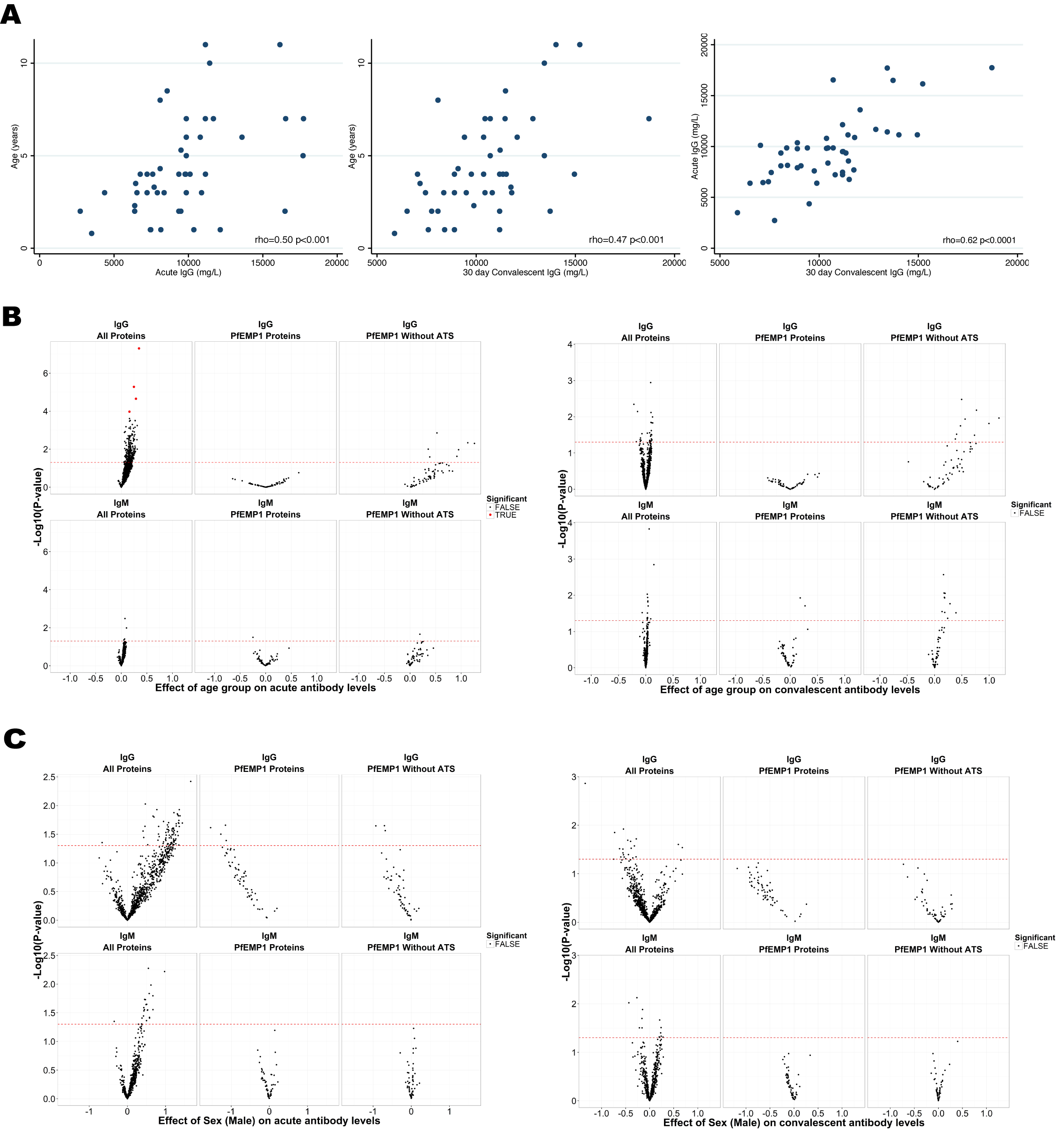

Supplement: Supplementary file 4 — Additional file 4. Assessment of P. falciparum and total Ig levels by age and sex. (a) Spearman correlations comparing age vs. acute total IgG level, age vs. 30-day convalescent total IgG level, and total IgG levels across timepoints show significant linear trends. (b) Volcano plots comparing the effect of age (> 5 years/≤ 5 years) on IgG, IgM seroreactivity to P. falciparum and PfEMP1 antigens indicates that for IgG in acute infection, age affects the magnitude of seroreactivity to a few P. falciparum antigens and a general non-significant trend for higher antibodies to all proteins in older children during acute infection (CM + UM total n = 48). (c) Volcano plots of inverse unadjusted P-values (y-axis) and linear regression effect estimates (x-axis) for comparing the effect of sex (male/female) on IgG, IgM seroreactivity to P. falciparum and PfEMP1 antigens indicate that sex does not have a significant effect on seroreactivity in our patient population in acute infection or convalescence, although non-significant trends for higher overall P. falciparum-specific IgG and IgM in males during acute infection and lower PfEMP1-specific IgG in males for both time points could be observed (CM + UM total n = 48). Dashed lines represent unadjusted P-value of 0.05. Points are highlighted in bold, red if significant after adjustment for the false discovery rate. [file 12936_2018_2323_MOESM4_ESM.tif]

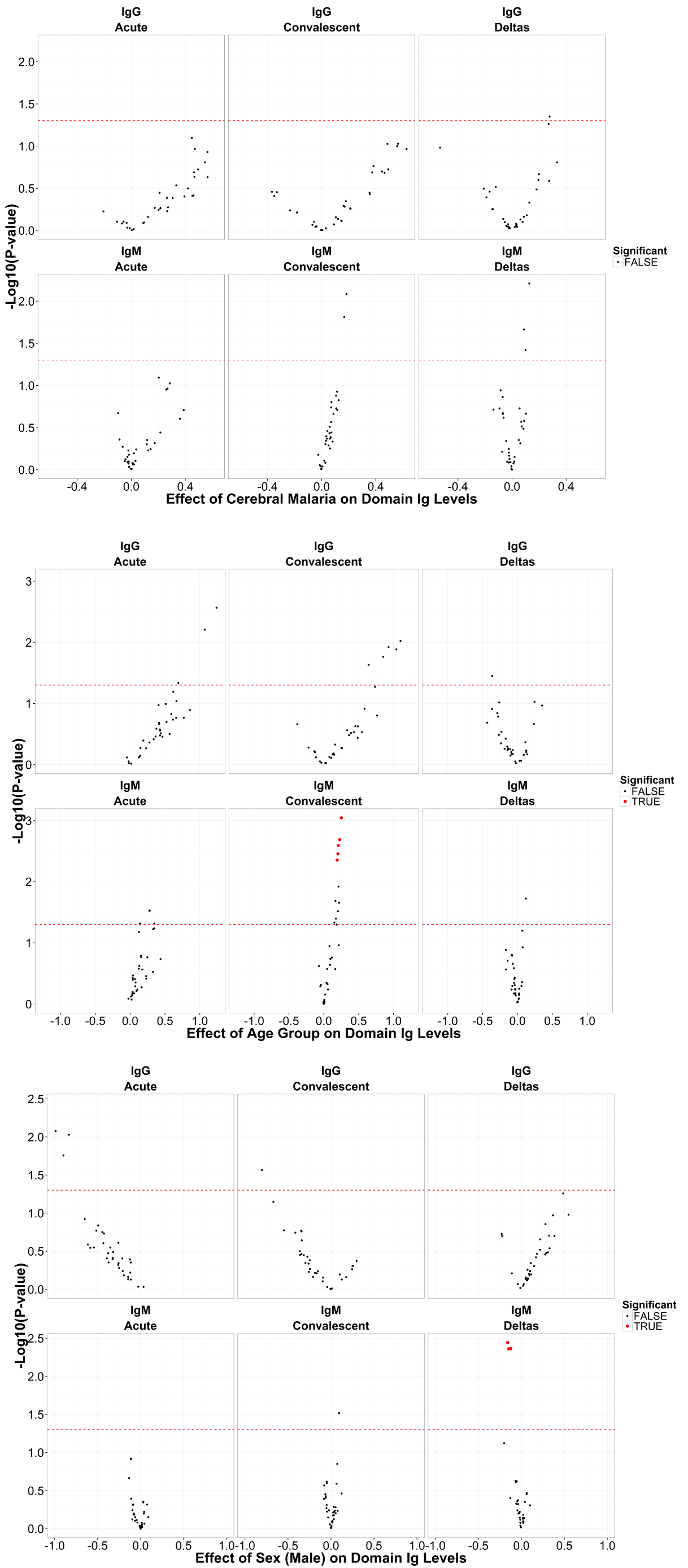

Supplement: Supplementary file 9 — Additional file 9. Additional exposure model covariates: the effect of cerebral malaria, age, and sex on PfEMP1 antibody level. Volcano plots of linear regression effect estimates (x-axis) for the independent variables (cerebral malaria, Ret + CM/UM; age, ≤ 5/> 5; sex, male/female) included in the exposure models generated for PfEMP1 expressed domains as determined by qRT-PCR of infected blood samples obtained during acute disease. (Total n = 48; CM n = 25; > 5 years of age n = 14; male n = 32). [file 12936_2018_2323_MOESM9_ESM.tif]
