# Supplementary material for: Convalescent Plasmodium falciparum-specific seroreactivity does not correlate with paediatric malaria severity or Plasmodium antigen exposure
Source: Malar J. 2018 Apr 25;17:178. doi: 10.1186/s12936-018-2323-4 (PMC5918990; doi:10.1186/s12936-018-2323-4)
Supplement: Supplementary file 5 — Additional file 5. Reactivity to markers of prior malaria exposure and antigens of interest in control populations. [file 12936_2018_2323_MOESM5_ESM.docx]

| **Table S2. Reactivity to markers of prior malaria exposure and antigens of interest in control populations** | | | | |
| --- | --- | --- | --- | --- |
| **Antigen/marker** | **Gene ID** | **Hyperimmune IgG R** | **Naïve IgG R** |  |
| Plasmodium exported protein (GEXP18) | PF3D7_0402400 | 5.20 | 1.89 |  |
| Exonuclease, putative | PF3D7_1106300 | 3.28 | 1.13 | Markers of |
| Erythrocyte membrane protein 1 (VAR) | PF3D7_0711700 | 5.76 | 4.25 | prior exposure |
| Erythrocyte membrane protein 1 (VAR) | PF3D7_0800300 | 5.92 | 2.69 |  |
| Heat shock protein 40, type II (HSP40) | PF3D7_0501100 | 5.00 | 0.42 | Helb et al. |
| Early transcribed membrane protein 4 (ETRAMP4) | PF3D7_0423700 | 4.59 | 3.02 | *PNAS*, 2015 |
| Acyl-coA synthetase (ACS5) | PF3D7_0731600 | 5.18 | 0.74 |  |
| PF70 protein (PF70) | PF3D7_1002100 | 5.46 | 3.21 |  |
|  |  |  |  |  |
| Circumsporozoite protein (CSP) | PF3D7_0304600 | 2.45 | 0.58 |  |
| Apical membrane antigen 1 (AMA1) | PF3D7_1133400 | 4.47 | 0.78 | Vaccine candidates & |
| Merozoite surface protein 1 (MSP1) | PF3D7_0930300 | 6.45 | 1.45 | antigens of interest |
| Merozoite surface protein 2 (MSP2) | PF3D7_0206800 | 5.74 | 2.64 |  |
| Erythrocyte binding antigen-175 (EBA175) | PF3D7_0731500 | 5.88 | 1.01 |  |
| Glutamate-rich protein (GLURP) | PF3D7_1035300 | 6.21 | 0.81 |  |
| Liver stage antigen 1 (LSA1) | PF3D7_1036400 | 6.11 | 2.38 |  |
| Liver stage antigen 3 (LSA3) | PF3D7_0220000 | 6.70 | 2.65 |  |
| **R, seroreactivity where R>1 indicates a seropositive response.** | |  |  |  |
