# Supplementary material for: Convalescent Plasmodium falciparum-specific seroreactivity does not correlate with paediatric malaria severity or Plasmodium antigen exposure
Source: Malar J. 2018 Apr 25;17:178. doi: 10.1186/s12936-018-2323-4 (PMC5918990; doi:10.1186/s12936-018-2323-4)
Supplement: Supplementary file 6 — Additional file 6. Seroreactivity to PfEMP1 antigens in pooled control serum vs. paediatric malaria cases. [file 12936_2018_2323_MOESM6_ESM.docx]

| **Table S3. Seroreactivity to PfEMP1 antigens in pooled control serum vs. acute pediatric malaria sera** | | | | | |
| --- | --- | --- | --- | --- | --- |
| **Binding phenotype** | **Gene.ID** | **Descriptions** | **Hyperimmune IgG** | **Naïve**  **IgG** | **UM + CM**  **acute** |
| **EPCR** | PF3D7_0400400 | **CIDRα1.1** | **1.01** | **-0.02** | **-0.02** |
|  | PF3D7_1150400 | **CIDRα1.4** | **1.21** | **0.07** | **0.89** |
|  | PF3D7_0800300 | **CIDRα1.6** | **1.84** | **1.60** | **0.80** |
|  | PF3D7_0425800 | **CIDRα1.6** | **1.12** | **0.27** | **0.48** |
|  | PF3D7_0600200 | **CIDRα1.8** | **1.06** | **0.12** | **0.28** |
| **ICAM-1** |  |  |  |  |  |
|  | PF3D7_1150400 | **DBLβ3-DBLβ6** | **2.81** | **0.23** | **0.51** |
|  | PF3D7_0425800 | **DBLβ3-DBLβ3** | **2.45** | **1.56** | **0.67** |
|  |  |  |  |  |  |
| **Rosetting** | PF3D7_1300300 | **CIDRδ1** | **3.42** | **1.60** | **1.72** |
|  | PF3D7_0800200 | **CIDRδ2** | **0.94** | **0.10** | **0.21** |
|  |  |  |  |  |  |
| **CD36** | PF3D7_0324900 | **CIDRα2.1** | **3.68** | **1.06** | **1.57** |
|  | PF3D7_0800100 | **CIDRα2.1** | **3.83** | **0.87** | **0.78** |
|  | PF3D7_0400100 | **CIDRα2.1** | **4.42** | **1.03** | **0.60** |
|  | PF3D7_0617400 | **CIDRα2.1** | **2.48** | **0.39** | **0.38** |
|  | PF3D7_1200100 | **CIDRα2.2** | **2.01** | **1.31** | **0.70** |
|  | PF3D7_0200100 | **CIDRα2.2** | **0.62** | **0.08** | **0.06** |
|  | PF3D7_0809100 | **CIDRα2.2** | **1.61** | **0.65** | **0.69** |
|  | PF3D7_1255200 | **CIDRα2.3** | **1.36** | **-0.16** | **0.13** |
|  | PF3D7_0712800 | **CIDRα2.4** | **1.62** | **0.18** | **0.32** |
|  | PF3D7_0300100 | **CIDRα2.4** | **1.23** | **-0.12** | **0.15** |
|  | PF3D7_0500100 | **CIDRα2.4** | **1.02** | **0.39** | **0.78** |
|  | PF3D7_1300100 | **CIDRα2.4** | **1.51** | **0.40** | **1.03** |
|  | PF3D7_1041300 | **CIDRα2.7** | **3.84** | **0.91** | **1.77** |
|  | PF3D7_0115700 | **CIDRα2.8** | **1.34** | **1.29** | **0.37** |
|  | PF3D7_0100100 | **CIDRα2.8** | **1.32** | **-0.06** | **0.14** |
|  | PF3D7_0808700 | **CIDRα3.1** | **2.81** | **1.06** | **1.88** |
|  | PF3D7_1000100 | **CIDRα3.1** | **3.07** | **0.95** | **1.3** |
|  | PF3D7_0712900 | **CIDRα3.1** | **0.13** | **-0.01** | **-0.03** |
|  | PF3D7_1240600 | **CIDRα3.1** | **2.07** | **0.44** | **0.07** |
|  | PF3D7_0937800 | **CIDRα3.1** | **2.26** | **0.76** | **1.49** |
|  | PF3D7_0412900 | **CIDRα3.1** | **1.34** | **0.32** | **0.30** |
|  | PF3D7_0712600 | **CIDRα3.1** | **3.01** | **0.55** | **1.01** |
|  | PF3D7_0712000 | **CIDRα3.1** | **1.27** | **0.16** | **0.14** |
|  | PF3D7_0833500 | **CIDRα3.1** | **2.84** | **0.08** | **1.01** |
|  | PF3D7_0632500 | **CIDRα3.2** | **2.11** | **0.31** | **1.78** |
|  | PF3D7_0420700 | **CIDRα3.2** | **2.02** | **0.46** | **0.10** |
|  | PF3D7_0412700 | **CIDRα3.2** | **0.75** | **0.10** | **0.24** |
|  | PF3D7_0808600 | **CIDRα3.2** | **1.47** | **0.33** | **0.36** |
|  | PF3D7_1100100 | **CIDRα3.2** | **1.90** | **0.66** | **0.47** |
|  | PF3D7_0900100 | **CIDRα3.4** | **1.36** | **0.19** | **0.78** |
|  | PF3D7_1219300 | **CIDRα3.4** | **1.13** | **0.19** | **0.21** |
|  | PF3D7_1373500 | **CIDRα3.4** | **0.89** | **0.08** | **0.24** |
|  | PF3D7_0733000 | **CIDRα3.4** | **0.24** | **-0.01** | **0.01** |
|  | PF3D7_0223500 | **CIDRα3.4** | **2.02** | **1.04** | **1.29** |
|  | PF3D7_1240400 | **CIDRα3.4** | **1.67** | **1.16** | **1.02** |
|  | PF3D7_0421100 | **CIDRα4** | **2.54** | **0.90** | **0.58** |
|  | PF3D7_0632800 | **CIDRα4** | **2.87** | **2.46** | **0.60** |
|  | PF3D7_1240300 | **CIDRα4** | **3.17** | **2.61** | **0.85** |
|  | PF3D7_0426000 | **CIDRα4** | **0.69** | **0.07** | **0.10** |
|  | PF3D7_1200400 | **CIDRα5** | **1.09** | **0.89** | **0.51** |
|  | PF3D7_0712400 | **CIDRα6** | **0.40** | **-0.03** | **0.06** |
| **R, seroreactivity where R>1 indicates a seropositive response. UM + CM, n=48; Median acute R depicted.** | | | | | |
