# Supplementary material for: Convalescent Plasmodium falciparum-specific seroreactivity does not correlate with paediatric malaria severity or Plasmodium antigen exposure
Source: Malar J. 2018 Apr 25;17:178. doi: 10.1186/s12936-018-2323-4 (PMC5918990; doi:10.1186/s12936-018-2323-4)
Supplement: Supplementary file 7 — Additional file 7. CIDR IgG responders at time of acute P. falciparum infection. [file 12936_2018_2323_MOESM7_ESM.docx]

| **Table S4. CIDR IgG responders at time of acute *P. falciparum* infection** | | | | |  |
| --- | --- | --- | --- | --- | --- |
|  |  |  |  |  |  |
| **Binding phenotype** | **Gene ID** | **CIDR domain** | **Responders** | **% UM Responders** | **% CM Responders** |
| **EPCR** |  |  |  |  |  |
|  | PF3D7_0400400 | CIDRα1.1 | 2 | 4% | 4% |
|  | PF3D7_1150400 | CIDRα1.4 | 20 | 35% | 48% |
|  | PF3D7_0800300 | CIDRα1.6 | 20 | 43% | 40% |
|  | PF3D7_0425800 | CIDRα1.6 | 12 | 17% | 32% |
|  | PF3D7_0600200 | CIDRα1.8 | 10 | 22% | 19% |
| **ICAM-1** |  |  |  |  |  |
|  | PF3D7_1150400 | DBLβ3 | 15 | 26% | 36% |
|  | PF3D7_0425800 | DBLβ3 | 20 | 35% | 48% |
| **Rosetting** |  |  |  |  |  |
|  | PF3D7_1300300 | CIDRδ1 | 33 | 61% | 76% |
|  | PF3D7_0800200 | CIDRδ2 | 6 | 17% | 8% |
| **CD36** |  |  |  |  |  |
|  | PF3D7_0324900 | CIDRα2.1 | 33 | 61% | 76% |
|  | PF3D7_0800100 | CIDRα2.1 | 22 | 48% | 44% |
|  | PF3D7_0400100 | CIDRα2.1 | 16 | 30% | 36% |
|  | PF3D7_0617400 | CIDRα2.1 | 12 | 13% | 36% |
|  | PF3D7_1200100 | CIDRα2.2 | 18 | 39% | 36% |
|  | PF3D7_0200100 | CIDRα2.2 | 0 | 0% | 0% |
|  | PF3D7_0809100 | CIDRα2.2 | 18 | 35% | 40% |
|  | PF3D7_0200100 | CIDRα2.2 | 39 | 78% | 84% |
|  | PF3D7_1255200 | CIDRα2.3 | 5 | 13% | 8% |
|  | PF3D7_0712800 | CIDRα2.4 | 11 | 17% | 28% |
|  | PF3D7_0300100 | CIDRα2.4 | 12 | 22% | 28% |
|  | PF3D7_0500100 | CIDRα2.4 | 19 | 35% | 44% |
|  | PF3D7_1300100 | CIDRα2.4 | 24 | 48% | 52% |
|  | PF3D7_1041300 | CIDRα2.7 | 35 | 65% | 80% |
|  | PF3D7_0115700 | CIDRα2.8 | 8 | 26% | 8% |
|  | PF3D7_0100100 | CIDRα2.8 | 3 | 9% | 4% |
|  | PF3D7_0808700 | CIDRα3.1 | 34 | 65% | 76% |
|  | PF3D7_1000100 | CIDRα3.1 | 27 | 43% | 68% |
|  | PF3D7_0712900 | CIDRα3.1 | 1 | 4% | 0% |
|  | PF3D7_1240600 | CIDRα3.1 | 5 | 13% | 8% |
|  | PF3D7_0937800 | CIDRα3.1 | 30 | 57% | 68% |
|  | PF3D7_0412900 | CIDRα3.1 | 11 | 26% | 20% |
|  | PF3D7_0712600 | CIDRα3.1 | 25 | 43% | 60% |
|  | PF3D7_0712000 | CIDRα3.1 | 5 | 13% | 8% |
|  | PF3D7_0833500 | CIDRα3.1 | 26 | 43% | 64% |
|  | PF3D7_0632500 | CIDRα3.2 | 34 | 74% | 68% |
|  | PF3D7_0420700 | CIDRα3.2 | 4 | 4% | 12% |
|  | PF3D7_0420900 | CIDRα3.2 | 3 | 9% | 4% |
|  | PF3D7_0711700 | CIDRα3.2 | 4 | 4% | 12% |
|  | PF3D7_0412700 | CIDRα3.2 | 10 | 26% | 16% |
|  | PF3D7_0808600 | CIDRα3.2 | 7 | 13% | 16% |
|  | PF3D7_1100100 | CIDRα3.2 | 15 | 26% | 36% |
|  | PF3D7_0421300 | CIDRα3.4 | 10 | 22% | 20% |
|  | PF3D7_0900100 | CIDRα3.4 | 17 | 39% | 32% |
|  | PF3D7_1219300 | CIDRα3.4 | 8 | 22% | 12% |
|  | PF3D7_1373500 | CIDRα3.4 | 6 | 17% | 8% |
|  | PF3D7_0733000 | CIDRα3.4 | 0 | 0% | 0% |
|  | PF3D7_0223500 | CIDRα3.4 | 30 | 61% | 64% |
|  | PF3D7_1240400 | CIDRα3.4 | 25 | 48% | 56% |
|  | PF3D7_0421100 | CIDRα4 | 11 | 17% | 28% |
|  | PF3D7_0632800 | CIDRα4 | 15 | 26% | 36% |
|  | PF3D7_1240300 | CIDRα4 | 22 | 52% | 40% |
|  | PF3D7_0426000 | CIDRα4 | 1 | 4% | 0% |
|  | PF3D7_1200400 | CIDRα5 | 10 | 13% | 28% |
|  | PF3D7_0712400 | CIDRα6 | 0 | 0% | 0% |
| **Responders, n (of 48 total). % UM of n=23. % CM of n=25.** | | |  |  |  |
