# Supplementary material for: Convalescent Plasmodium falciparum-specific seroreactivity does not correlate with paediatric malaria severity or Plasmodium antigen exposure
Source: Malar J. 2018 Apr 25;17:178. doi: 10.1186/s12936-018-2323-4 (PMC5918990; doi:10.1186/s12936-018-2323-4)
Supplement: Supplementary file 8 — Additional file 8. Reactivity to head structure/CIDR or ICAM-binding domain in unexposed cases. [file 12936_2018_2323_MOESM8_ESM.docx]

| **Table S5. Reactivity to head structure/CIDR or ICAM-1 binding domain in unexposed cases** | | | | | |  |  | |
| --- | --- | --- | --- | --- | --- | --- | --- | --- |
| **Binding** | **Pf1000 array** | **Binding** | **Unexposed** | **Acute Responders** | **New Responders** | **Non Responders** |  | |
| **phenotype** | **gene ID** | **domain** | **(n of 48)** | **% (n)** | **% (n)** | **% (n)** |  | |
| **EPCR** |  |  |  |  |  |  |  | |
|  | PF3D7_0400400 | CIDRα1.1 | 22 | 5% (1) | 9% (2) | 86% (19) |  | |
|  | PF3D7_1150400 | CIDRα1.4* | 46 | 44% (20) | 15% (7) | 41% (19) |  | |
|  | PF3D7_0800300 | CIDRα1.6 | 39 | 46% (18) | 3% (1) | 51% (20) |  | |
|  | PF3D7_0425800 | CIDRα1.6* | 39 | 28% (11) | 10% (4) | 62% (24) |  | |
| **ICAM-1^** |  |  |  |  |  |  |  | |
|  | PF3D7_1150400 | DBLβ3 | 17 | 41% (7) | 24% (4) | 35% (6) |  | |
|  | PF3D7_0425800 | DBLβ3 | 17 | 59% (10) | 18% (3) | 24% (4) |  | |
| **Rosetting** |  |  |  |  |  |  |  | |
|  | PF3D7_1300300 | CIDRδ1 | 36 | 69% (25) | 6% (2) | 25% (9) |  | |
|  | PF3D7_0800200 | CIDRδ2 | 36 | 17% (6) | 0% (0) | 83% (30) |  | |
| **CD36** |  |  |  |  |  |  |  | |
|  | PF3D7_1200100 | CIDRα2.2 | 40 | 35% (14) | 5% (2) | 60% (24) |  | |
|  | PF3D7_0200100 | CIDRα2.2 | 40 | 0% (0) | 2% (1) | 98% (39) |  | |
|  | PF3D7_0809100 | CIDRα2.2 | 40 | 35% (14) | 12% (5) | 53% (21) |  | |
|  | PF3D7_0200100 | CIDRα2.2 | 40 | 80% (32) | 15% (6) | 5% (2) |  | |
|  | PF3D7_1255200 | CIDRα2.3 | 16 | 6% (1) | 6% (1) | 88% (14) |  | |
|  | PF3D7_1041300 | CIDRα2.7 | 16 | 75% (12) | 12% (2) | 12% (2) |  | |
|  | PF3D7_0808700 | CIDRα3.1 | 34 | 79% (27) | 12% (4) | 9% (3) |  | |
|  | PF3D7_1000100 | CIDRα3.1 | 34 | 59% (20) | 9% (3) | 32% (11) |  | |
|  | PF3D7_0712900 | CIDRα3.1 | 34 | 3% (1) | 3% (1) | 94% (32) |  | |
|  | PF3D7_1240600 | CIDRα3.1 | 34 | 12% (4) | 3% (1) | 85% (29) |  | |
|  | PF3D7_0937800 | CIDRα3.1 | 34 | 62% (21) | 18% (6) | 21% (7) |  | |
|  | PF3D7_0412900 | CIDRα3.1 | 34 | 29% (10) | 3% (1) | 68% (23) |  | |
|  | PF3D7_0712600 | CIDRα3.1 | 34 | 59% (20) | 12% (4) | 29% (10) |  | |
|  | PF3D7_0712000 | CIDRα3.1 | 34 | 15% (5) | 6% (2) | 79% (27) |  | |
|  | PF3D7_0833500 | CIDRα3.1 | 34 | 59% (20) | 12% (4) | 29% (10) |  | |
|  | PF3D7_0632500 | CIDRα3.2 | 34 | 73% (25) | 15% (5) | 12% (4) |  | |
|  | PF3D7_0420700 | CIDRα3.2 | 34 | 12% (4) | 0% (0) | 88% (30) |  | |
|  | PF3D7_0420900 | CIDRα3.2 | 34 | 9% (3) | 0% (0) | 91% (31) |  | |
|  | PF3D7_0711700 | CIDRα3.2 | 34 | 12% (4) | 0% (0) | 88% (30) |  | |
|  | PF3D7_0412700 | CIDRα3.2 | 34 | 24% (8) | 3% (1) | 73% (25) |  | |
|  | PF3D7_0808600 | CIDRα3.2 | 34 | 15% (5) | 0% (0) | 85% (29) |  | |
|  | PF3D7_1100100 | CIDRα3.2 | 34 | 32% (11) | 6% (2) | 62% (21) |  | |
|  | PF3D7_0421300 | CIDRα3.4 | 22 | 27% (6) | 9% (2) | 64% (14) |  | |
|  | PF3D7_0900100 | CIDRα3.4 | 22 | 36% (8) | 9% (2) | 55% (12) |  | |
|  | PF3D7_1219300 | CIDRα3.4 | 22 | 18% (4) | 5% (1) | 77% (17) |  | |
|  | PF3D7_1373500 | CIDRα3.4 | 22 | 14% (3) | 0% (0) | 86% (19) |  | |
|  | PF3D7_0733000 | CIDRα3.4 | 22 | 0% (0) | 0% (0) | 100% (22) |  | |
|  | PF3D7_0223500 | CIDRα3.4 | 22 | 77% (17) | 9% (2) | 14% (3) |  | |
|  | PF3D7_1240400 | CIDRα3.4 | 22 | 55% (12) | 18% (4) | 27% (6) |  | |
| **Unexposed, domain absent/not amplified in acute infection; Responders: Acute, reactive in acute infection;**  **New, reactive only in convalescence; Non, not reactive.** | | | | | | | | |
| **^Exposure status available for n=42; *Head structure/CIDR domains followed by ICAM-1 DBLβ3 domain (CIDRα1-DBLβ).** | | | | | | | |  |
